# Targeted Resequencing of the Pericentromere of Chromosome 2 Linked to Constitutional Delay of Growth and Puberty

**DOI:** 10.1371/journal.pone.0128524

**Published:** 2015-06-01

**Authors:** Diana L. Cousminer, Jaakko T. Leinonen, Antti-Pekka Sarin, Himanshu Chheda, Ida Surakka, Karoliina Wehkalampi, Pekka Ellonen, Samuli Ripatti, Leo Dunkel, Aarno Palotie, Elisabeth Widén

**Affiliations:** 1 Institute for Molecular Medicine Finland (FIMM), University of Helsinki, Helsinki, Finland; 2 Public Health Genomics Unit, Department of Chronic Disease Prevention, National Institute for Health and Welfare, Helsinki, Finland; 3 Diabetes Prevention Unit, National Institute for Health and Welfare, Helsinki, Finland; 4 Children’s Hospital, Helsinki University Central Hospital and University of Helsinki, Helsinki, Finland; 5 Department of Public Health, Hjelt Institute, University of Helsinki, Helsinki, Finland; 6 Wellcome Trust Sanger Institute, Hinxton, Cambridge, United Kingdom; 7 Centre for Endocrinology, William Harvey Research Institute, Barts and the London School of Medicine and Dentistry, London, United Kingdom; 8 The Medical and Population Genomics Program, Broad Institute of MIT and Harvard, Cambridge, MA, United States of America; Institut Jacques Monod, FRANCE

## Abstract

Constitutional delay of growth and puberty (CDGP) is the most common cause of pubertal delay. CDGP is defined as the proportion of the normal population who experience pubertal onset at least 2 SD later than the population mean, representing 2.3% of all adolescents. While adolescents with CDGP spontaneously enter puberty, they are at risk for short stature, decreased bone mineral density, and psychosocial problems. Genetic factors contribute heavily to the timing of puberty, but the vast majority of CDGP cases remain biologically unexplained, and there is no definitive test to distinguish CDGP from pathological absence of puberty during adolescence. Recently, we published a study identifying significant linkage between a locus at the pericentromeric region of chromosome 2 (chr 2) and CDGP in Finnish families. To investigate this region for causal variation, we sequenced chr 2 between the genomic coordinates of 79–124 Mb (genome build GRCh37) in the proband and affected parent of the 13 families contributing most to this linkage signal. One gene, *DNAH6*, harbored 6 protein-altering low-frequency variants (< 6% in the Finnish population) in 10 of the CDGP probands. We sequenced an additional 135 unrelated Finnish CDGP subjects and utilized the unique Sequencing Initiative Suomi (SISu) population reference exome set to show that while 5 of these variants were present in the CDGP set, they were also present in the Finnish population at similar frequencies. Additional variants in the targeted region could not be prioritized for follow-up, possibly due to gaps in sequencing coverage or lack of functional knowledge of non-genic genomic regions. Thus, despite having a well-characterized sample collection from a genetically homogeneous population with a large population-based reference sequence dataset, we were unable to pinpoint variation in the linked region predisposing delayed puberty. This study highlights the difficulties of detecting genetic variants under linkage regions for complex traits and suggests that advancements in annotation of gene function and regulatory regions of the genome will be critical for solving the genetic background of complex phenotypes like CDGP.

## Introduction

In healthy adolescents, the timing of pubertal onset varies widely. Constitutional delay of growth and puberty (CDGP) is defined as the absence of pubertal signs 2 SD later than the population mean for pubertal onset [1], and represents 2.3% of the normal population at the extreme late end. While these adolescents achieve normal pubertal growth and development spontaneously [2], they are at risk for short adult stature [3–6], decreased bone mineral density [7], and psychological distress [8]. Furthermore, distinguishing adolescents with normal pubertal delay from those who have pubertal absence requiring medical intervention (i.e., congenital hypogonadotropic hypogonadism, CHH) is challenging due to the lack of a definitive diagnostic test for clinical use [9].

Pubertal onset is strongly regulated by genetic factors, indicated by the high heritability (50–80%) between twins and in families [10]. At the tail ends of a normal population distribution, extreme phenotypes are likely to be driven by an oligogenic component made up of one or a few genetic variants of large effect size. To date, however, only a handful of genes have been found which harbor clinically relevant variation in adolescents with CDGP, and more than 99% of cases remain molecularly unexplained [11]. A homozygous partial loss-of-function (LoF) mutation in *GNRHR* was found in two brothers, one with CDGP and one with CHH [12], and another heterozygous mutation was found in one male with CDGP, while the cause of pubertal delay in the remaining 145 cases in the study remained unexplained [13]. Of 50 CDGP patients investigated for mutations in *TAC3* and *TAC3R*, only one mutation in a single patient was found in the latter gene [14]. Finally, 5 point mutations in 5 CDGP subjects were found in *GHSR* out of a total of 31 patients who were analyzed [15]. On the other hand, many genes are now known to influence the timing of puberty at the population level [16–18], and a recent study showed that some genes underlying rare Mendelian disorders of puberty also harbor more common variants subtly influencing normal variation in the timing of puberty [19].

Recently, studies of multiply affected Finnish CDGP families showed that 74% of unilineal pedigrees had apparent autosomal dominant inheritance of a susceptibility locus [20], and further investigation revealed a region of linkage at the pericentromeric region of chr 2 at 2p13-2q13 potentially harboring variation predisposing to pubertal delay [21]. In the present study, we followed up on this linkage signal by resequencing the complete genomic region of chr 2 from 79–124 Mb in the 13 probands and their affected parent who contributed the most to the linkage finding. Since by definition CDGP is present in around 2% of the general population, the predisposing variant is expected to be present in the Finnish population at a low frequency (≤ 5%). We therefore investigated low-frequency variation in the region, focusing on coding variation in any gene and regulatory variation in functionally relevant candidate genes, but were unable to detect a susceptibility variant that transmitted along with pubertal delay in these families.

## Materials and Methods

Initially, genome-wide genotyping was performed using commercially available genotyping chips to determine genome-wide characteristics of the 13 families contributing the most to the linkage finding, including their degree of relatedness to each other and their genetic relationship compared to population controls. Next, we sequenced the region falling under the linkage peak on chr 2 between 79–124 Mb. After variant filtering, we looked for shared exonic variants or different variants falling in or near the same gene. We also looked at regulatory regions for genes that were functional candidates for involvement in growth and puberty.

### Study subjects

The identification of probands and ascertainment of family members have been previously described [21]. Briefly, patients referred to pediatric specialists due to pubertal delay were identified from hospital records from Helsinki, Kuopio, Tampere, and Turku University Hospitals as well as two municipal hospitals in southern Finland. Patients were included if they fulfilled diagnostic criteria for CDGP, defined as the onset of pubertal development 2 SD later than the population mean (later than 13.5 years in boys for Tanner genital stage II and later than 13.0 years in girls for Tanner breast stage II), and if other causes of pubertal delay (chronic illness or CHH) could be excluded. Furthermore, growth charts were examined, and the pubertal growth spurt also began 2 SD later than the population average (age at takeoff of pubertal growth later than 13.8 years in boys and 12.2 years in girls, and age at peak height velocity (PHV) later than 15.6 years in boys and 13.7 years in girls) [22].

Family members of probands were ascertained via invitation letters and structured interviews. Archived height measurements were used to draw growth charts, and the timing of the pubertal growth spurt was used to determine the timing of puberty when possible. Because the delay in puberty may be variable in penetrance, family members were considered to have CDGP if the age at takeoff or PHV of the pubertal growth spurt occurred more than 1.5 SD later than the mean, or if they attained adult height at later than 18 years for males or 16 years for females [21].

**Fig 1:**
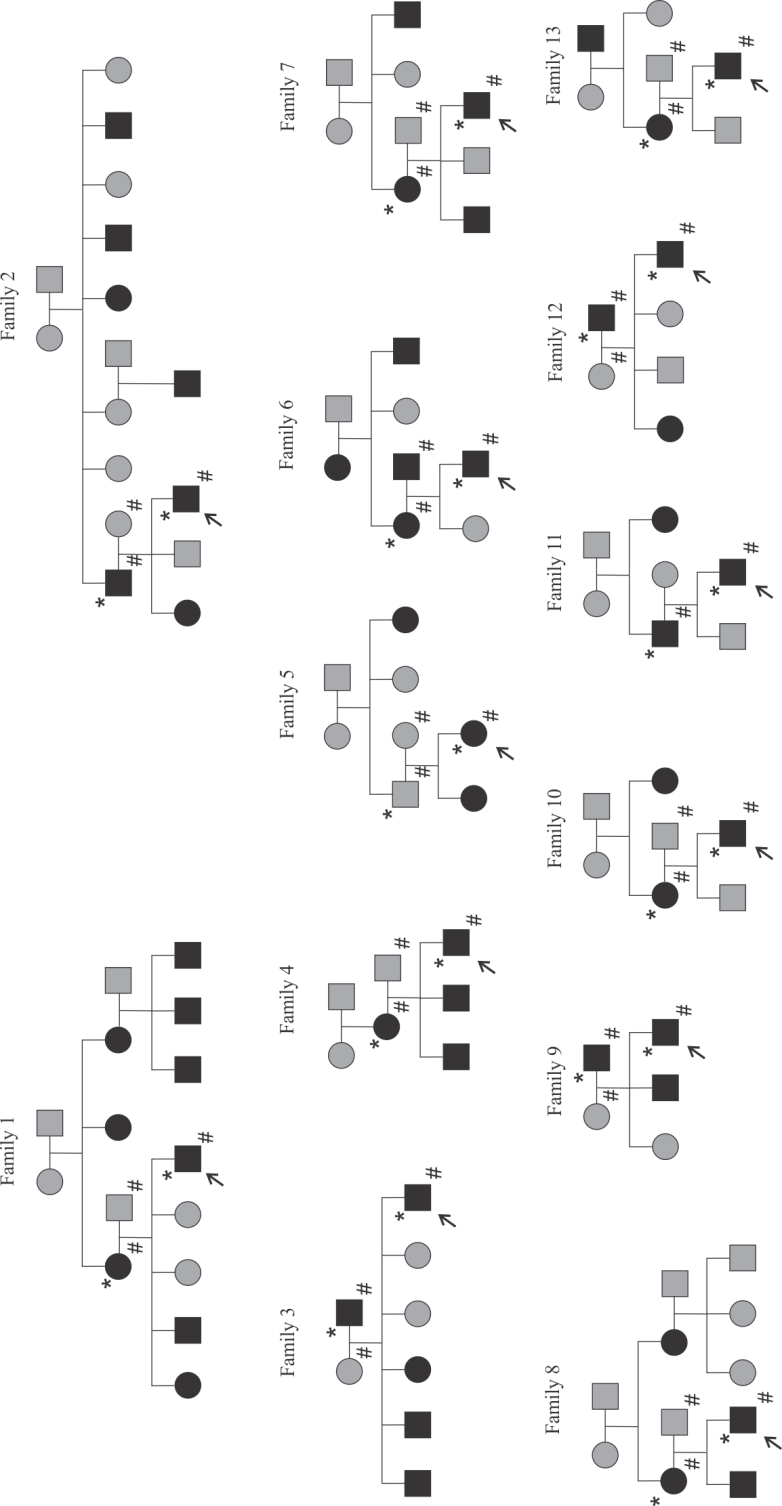
Pedigrees of the 13 families included in the study. Circles represent females and squares are males. Filled circles are classified as affected with CDGP, while shaded circles are unknown or do not fulfill the criteria for CDGP. The proband from each family is marked with an arrow. Individuals with an asterisk (*) have been sequenced at the pericentromere of chr 2. All probands and both of their parents were genotyped (denoted with the symbol #) except family 11, in which only the proband and affected parent were genotyped.

In the current study, we included the proband and his or her parents from the top 13 families (**Fig 1**) contributing the most to the previously published linkage signal based on family-specific LOD scores in the linkage region on chr 2p13-2q13 [21]. All participants and/or his or her guardian gave written informed consent, and the study design and sample collection were approved by the Ethics Committee for Pediatrics, Adolescent Medicine, and Psychiatry of the Hospital District of Helsinki and Uusimaa.

### Control samples

Whole-genome genotyped samples from the neurological services at the Kuopio University Hospital and the Helsinki University Hospital were originally collected as controls for a study on intracranial aneurysm (IA). 730 controls (pre-quality control) were collected from anonymous Finnish patients who gave blood for causes unrelated to IA, and genomic DNA was extracted from these blood samples. Genotypes were ascertained by the FIMM Technology Centre (Helsinki, Finland) using Illumina HumanCNV370-duo chips [23].

Additionally, several sets of publicly available control samples were used to ascertain the population frequencies of sequenced variants. These included the complete 1000 Genomes (1000G) reference set as well as the Finnish population-specific data from the 1000G reference (FIN-1000G; www.1000genomes.org/).

Finally, we utilized a publicly available set of Finnish exome sequences (*N* = 3,325) from the Sequencing Initiative Suomi (SISu) project (http://sisu.fimm.fi) to confirm population-specific allele frequencies of exonic variants. In the SISu project, whole-exome sequencing of Finnish individuals was done at the Wellcome Trust Sanger Institute (Cambridge, UK) using the Illumina platform. Variant recalling was performed jointly with ~23,000 exomes (~26,000 in total) from other studies at The Broad Institute (Cambridge, MA, USA). Variants that failed quality control filtering were removed as well as SNPs that were not within the bait regions of the exome capture kit. Genotypes with a quality metric below 20 were set to missing. Sample-wise quality control was performed after the removal of poor quality SNPs and individual genotypes. Variants with a call rate below 90% across all samples were also removed.

### Genome-wide genotyping

Genotyping of the proband and both parents (trios) of the 13 families that contributed the most to the previous linkage finding was performed on the Illumina Infinium platform at the FIMM Technology Centre (Helsinki, Finland) to determine genome-wide characteristics of these samples. Eight samples (the two top-scoring trios plus two probands from additional families) were genotyped using the HD HumanCNV370-Quad Beadchip, and thirty samples (eight trios, the parents of the probands that were genotyped on the HumanCNV370 chip, plus one additional proband and their affected parent) were genotyped with the Human610-Quad BeadChip. Genotype calls were made with BeadStudio Genotyping Module *v3*.*3*.*7* and reviewed manually. SNPs with a success rate of < 95% were excluded. Genotypes were retrieved for markers spanning all autosomes and the X chromosome. Genotyping was done in three batches, with average marker-wise success rates of 0.96, 0.93, and 0.97, respectively (**S1 Table**).

### Whole-genome analyses

We explored the general degree of relatedness among the 13 probands and compared their genetic variance against a population-based sample of random individuals (the IA controls) from roughly the same geographical region in Finland. First, PLINK [24] was used to calculate genome-wide identity-by-descent (IBD) among the 13 probands. Additionally, the pairwise inbreeding coefficient (F) was calculated in PLINK to confirm that there were no undetected close relationships between the probands’ parents.

**Fig 2:**
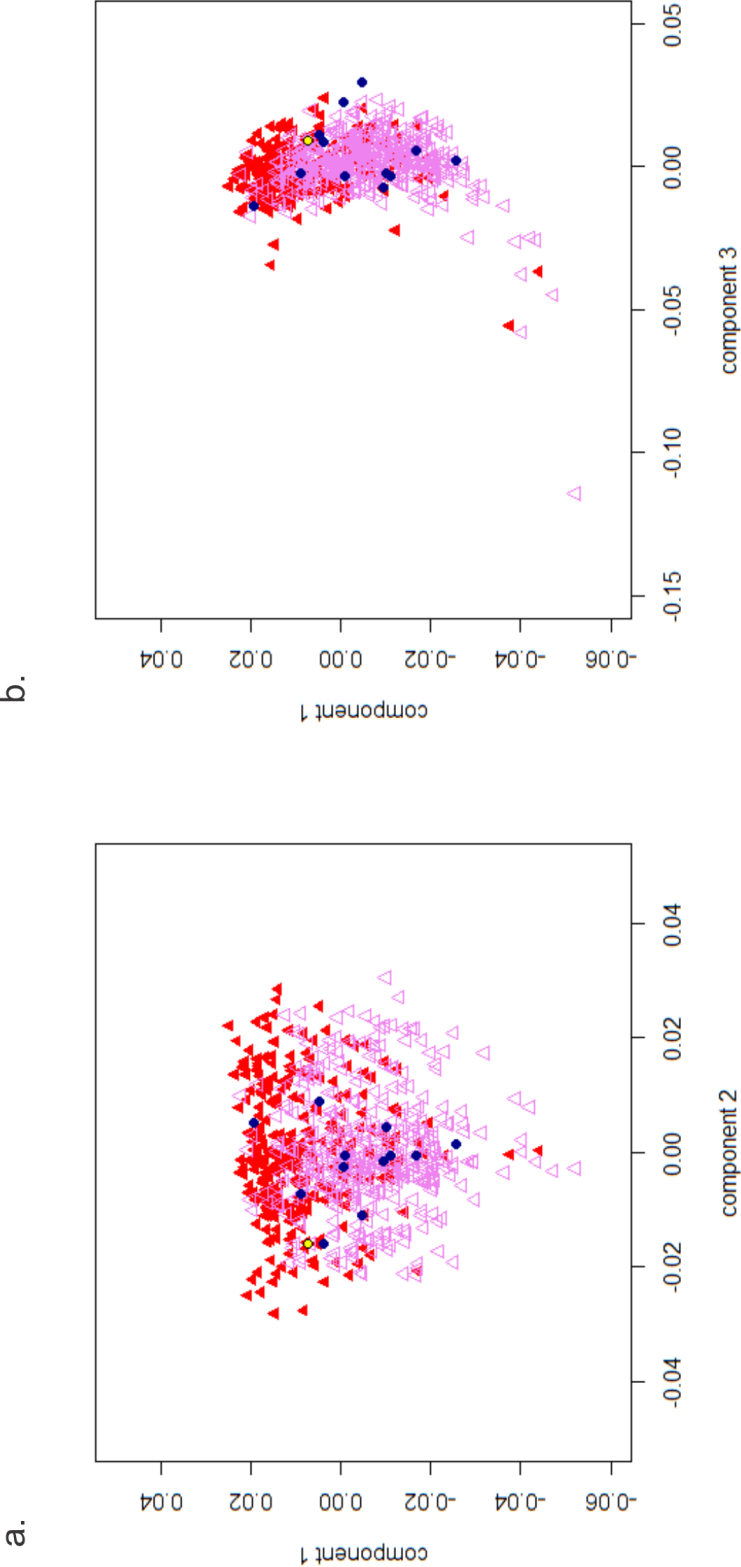
Multidimensional scaling (MDS) plots. Each point represents an individual genome. Red triangles are individuals from the Kuopio region of Finland while violet triangles are individuals from Helsinki. Each blue circle represents a proband from each of the 13 CDGP families. The yellow circle is the proband from Family 1. Panel A shows the relationship between principal components (PCs) 1 and 2, which explain most of the genetic variation. Panel B shows PC1 versus PC3, which appear to mimic a geographical northeast to southwest axis.

To better visualize the genomic variance between the CDGP probands and their relationship on a background of IA control subjects, the proportion of alleles shared identity-by-state (IBS) between all pairs of individuals was computed, and the first three principal components (PCs) were extracted. Multidimensional scaling (MDS) plots (**Fig 2**) were drawn using R *v*.*2*.*15*.*1* (www.r-project.org/).

### Targeted resequencing

Targeted resequencing of the pericentromeric region (79,171,971–124,250,162 Mb; genome build GRCh37) of chr 2 was performed in the proband and suspected affected parent of 13 families (*N* = 26). For library preparation, 3 μg of genomic DNA was processed according to the NEBNext DNA Sample Prep protocol (New England BioLabs, Ipswich, MA, USA). Subsequent target enrichment capture was performed using the NimbleGen SeqCap EZ Choice solution-based capture protocol. Sequencing was done on the Illumina HiSeq2000 platform with 100 bp paired-end reads. The variant calling, sequencing alignment, SNP calling, and indel calling have been previously described [25]. In brief, we used a variant calling pipeline (VCP) that was developed in-house for quality control, short read alignment, and variant calling and annotation. The sequencing and bioinformatics pipelines were performed at the Technology Centre at FIMM (Helsinki, Finland).

### Shared variant analyses

We prioritized the analysis of variants falling within or near known genes that were annotated with frequency information from the global 1000G dataset (MAF < 5%) and were transmitted from the affected parent to the proband. Additionally, variants with a MAF of over 5% in the FIN-1000G data were excluded. We also included any variants with no annotated frequency information into the analysis. Only variants located within the bait region of chr2:79–124 Mb were considered. We further prioritized variants with predicted consequences affecting protein structure (nonsynonymous, splice site, frameshift or premature stop codons) or within untranslated regions upstream or downstream of the gene (5’ or 3’ UTRs) or in introns. For many analyses, variants in family 1 were prioritized since this is a large pedigree of 15 individuals, 9 of whom were affected, that contributed the most to the linkage finding (**Fig 1**) [21].

The variant analysis was divided into three stages. We first examined whether family 1 shared identical variants or different variants annotated to the same gene with known 1000G frequencies of < 5% with any of the other families (**S2 Table**). Only genes with variants in 5 or more families are reported.

Secondly, we repeated the first analysis for variants with unknown frequency. Since we did not have frequency information to use as a filter, we prioritized variants found in family 1 (**S3 Table**).

Finally, we considered genic variants transmitted from either parent because it was not always possible to exclude bilineal inheritance. Thus, we also extracted variants transmitted from either parent annotated at < 5% in 1000G and looked for identical variants or different variants in the same gene that were shared by family 1 and at least 4 others (**S4 Table**).

Variants of interest were followed up by examining their minor allele frequencies in American (Exome Variant Server; http://evs.gs.washington.edu/EVS/) and Finnish (FIN-1000G and SISu) populations to confirm their rarity. Subsequently, we investigated the potential deleteriousness of these variants by looking at their SIFT [26] and PolyPhen (Polymorphism Phenotyping; http://genetics.bwh.harvard.edu/pph2/) [27] scores using the Variant Effect Predictor [28] (http://useast.ensembl.org/info/docs/tools/vep/index.html) and their RegulomeDB (http://regulome.stanford.edu/index) score. SIFT and PolyPhen predict whether a codon which alters the amino acid affects protein structure and function, while RegulomeDB identifies DNA features and regulatory elements in intergenic regions and assigns them a score which reflects their predicted ability to disrupt transcription factor binding.

### Candidate gene analyses

Alternatively to a protein-coding or splicing alteration, changes in important regulatory element sequences affecting gene expression could be another way a variant would impart a delay in pubertal growth and timing. To investigate the regulatory elements surrounding a set of plausible candidate genes, we downloaded genes located on chromosome 2 between 79–124 Mb which were annotated with appropriate gene ontology (GO) terms using Ensemble Biomart (http://www.ensembl.org/biomart/martview/). A complete list of GO terms queried can be found in **S5 Table**, and the resulting candidate list in **S6 Table**. The genes on this list were then manually queried from AceView (http://www.ncbi.nlm.nih.gov/IEB/Research/Acembly/) and PubMed (http://www.ncbi.nlm.nih.gov/pubmed) for additional evidence of involvement in puberty, resulting in a short list of the best functional candidates (*GLI2*, *PAX8*, *INHBB*, *KDM3A*, *NPHP1*, *BCL2L11*, *MERTK*, *IL1B*; **S6 Table**, genes marked in bold). Genes were only included in the list of the best candidates if they had functional evidence of involvement in both reproduction and growth, or direct involvement in the hypothalamic-pituitary-gonadal (HPG) axis.

We then investigated possible regulatory regions surrounding these 8 best functional candidate genes. According to a recent study, genetic variants which influence the expression of nearby genes center around the transcription start site of the gene, and cluster within 200 kb of the gene [29]. Thus, for this analysis, we looked at transmitted variants within 400 kb around these genes (200 kb from the gene’s start and terminus; **S7 Table**). To see if shared variants in these regions potentially affected protein regulation by altering transcription factor binding sites, we queried RegulomeDB (**S8 Table**), as well as checking for eQTLs potentially linking variants with nearby gene expression using the NCBI eQTL Browser (http://www.ncbi.nlm.nih.gov/projects/gap/eqtl/index.cgi).

Finally, a recent genome-wide association study pinpointed a variant in the linkage region associated with normal variation in age at menarche (rs6758290 at 105.8 Mb) [19]. Since this same study found enrichment of common variants associated with menarche in genes underlying rare Mendelian pubertal disorders, we investigated whether low-frequency variants in LD with rs6758290 might show evidence for involvement in pubertal delay in our CDGP families. While there are few variants in LD with this marker in European populations, the LD block (r^2^ ≥ 0.1) extends approximately 500 kb upstream and 300 kb downstream from rs6758290, with the majority of variants in high LD (r^2^ > 0.6) falling within 37 kb at or upstream of *GPR45* (**S1 Fig**). Thus, we extracted all variants from 105,364,800–106,164,800 (GRCh37) along chromosome 2 and looked for any transmitted shared variants (**S9 Table**). We also looked up the RegulomeDB score for all shared non-exonic variants to see if any had the potential to affect transcription factor binding.

### Follow-up sequencing in additional CDGP cases

The presence of low-frequency variants in two sets of three consecutive *DNAH6* exons (exons 23, 24, 25, 46, 47 and 48) in probands with CDGP (*N* = 135) was assessed by Sanger sequencing at the FIMM Technology Centre’s Sequencing Laboratory (FIMM SeqLab). The samples were amplified using Thermo Scientific DreamTaq Green PCR Master Mix (2X) (Thermo Fisher Scientific Inc, Waltham, MA) according to the manufacturer’s instructions. The primer sequences were designed with OligoArchitect Online v4.0 (Sigma Aldrich, St Louis, MO). Corresponding oligonucleotides were ordered from Sigma Aldrich. The primer sequences can be found in **S10 Table.** Cycling conditions were as follows: 95°C for 1 min, 35 cycles of 95°C for 30s, 52°C for 25s, and 72°C for 30s, followed by 72°C for 10 min. The samples were purified with ExoSAP-IT (Affymetrix, Santa Clara, CA, USA) according to instructions, and were then capillary-sequenced by the Genome Analyzer II (Illumina, San Diego, CA, USA) platform. Sequence analysis was performed with novoSNP3.0.1 [30]. The reference sequence was downloaded from the UCSC genome browser (hg19) [31], and allele frequencies were compared with the SISu Finnish population frequencies.

### Simulation analyses

There were six low-frequency variants found in *DNAH6* which had consequences on the protein’s structure (one stop codon and five nonsynonymous amino acid changes). These six variants were found in 10 out of 13 of the CDGP probands. With access to a large set of population-specific sequences from the SISu exome sequence set, we were able to investigate the probability of this occurrence. To do this, we looked at the approximately 5,000 Finnish exomes from the SISu data that are part of the ExAC browser (Exome Aggregation Consortium (ExAC), Cambridge, MA (URL: http://exac.broadinstitute.org) [Dec, 2014]). In total, there were 82 variants in this gene (79 SNPs and 3 indels). Running the Variant Effect Predictor (VEP; http://grch37.ensembl.org/Homo_sapiens/Tools/VEP) on these variants resulted in 29 nonsynonymous variants and 1 stop gain variant. Many of these individuals had missing genotypes for one or more loss of function (LoF) or nonsynonymous variants, so they were removed (final *N* = 2,028). One variant had a high alternate allele frequency (0.96), so this variant was flipped. We then ran simulation analyses by randomly choosing 13 individuals and counting how many times 10 or more of these individuals contained at least one of the 30 nonsynonmous or LoF variants. We ran this analysis twice, first with 10,000 iterations and second with 100,000 iterations. A summary of the read-depth and genotyping quality scores for the SISu and CDGP sequences can be found in **S11 Table**.

## Results

### Genome-wide genotyping shows that CDGP probands are not closely related

Genome-wide analyses on the genotyped data were done to identify genetic outliers falling outside the Finnish population as well as detect any previously unidentified close relationships among the families contributing to the linkage peak. Analysis of IBD aimed at detecting individuals who appeared more similar to each other than would be expected in a random population. The IBD estimate, as in the proportion of alleles predicted to be shared by descent from a common ancestor, for all pairwise combinations of affected probands was between 0.05–0.08 (0–0.08 in the unselected IA controls; 8 control pairs had values from 0.09–0.5 and thus one member of each pair was excluded from further analyses), and the F inbreeding coefficient was -0.03 to 0.02 (-0.06 to 0.1 in controls). These values indicate that the affected probands are not more closely related to each other than expected, nor is there a high probability of consanguinity among their recent ancestors.

The first three PCs were calculated based on the genotype data and used to create a matrix for MDS against the IA control samples (**Fig 2**). Interestingly, the first versus third PC mimics a geographical northeast to southwest axis, with individuals from the Kuopio region at the upper part of the graph, and individuals from Helsinki spread towards the lower part. The Finnish controls heavily overlap, in concordance with the post-World War II migration of Finns into the capital region [32]. Although a couple of the probands lie close to one another, in general they all fit homogenously within the background of controls from the Helsinki and Kuopio regions of Finland. Altogether, we conclude that none of the probands were either outliers or more closely related than expected.

### Targeted resequencing covered the targeted region successfully

Targeted resequencing of chr2 79–124 Mb was performed in 13 affected probands and their suspected affected parent (*N* = 26). The parent classified as affected was equally the mother (*N* = 7) or the father (*N* = 6) of the proband (**Fig 1**). Mean coverage depth in the target region across all samples was 60.6X (protein-coding regions 41X) with an average of 44,826,375 reads per sample. On average, 98.2% of reads aligned to the reference genome. In the targeted region, 82.2% of annotated exons (Ensembl protein-coding genes, *N* = 229) were covered at least 10X.

### Shared variant analyses revealed predicted protein-altering low-frequency variants in DNAH6

Low-frequency variants following the expected transmission pattern were found in three genes: *DNAH6*, *KDM3A*, and *LINC01102* (**S2 Table**). Low-frequency variants inherited from either parent were found within the gene *DDX18* (**S4 Table**), but some of these turned out to be fairly common in the Finnish population (MAF ~0.18). Overall, only *DNAH6* (Dynein, Axonemal, Heavy Chain 6), contained variants that met frequency and sharing criteria and had predicted protein-altering consequences. *DNAH6* harbored 6 variants in the top 13 families at low frequency with predicted stop or missense codons (**Table 1**). All 6 variants were present at less than ~5% in the Finnish population. In family 1, rs184604697 was transmitted from the affected parent to the proband and resulted in a premature stop codon (c.7689C>A, p.Tyr2563Ter). Two of the other 5 variants shared by other families were predicted to result in changes that would be damaging or deleterious by SIFT and/or PolyPhen, although these variants were equally transmitted from the affected parent or the alternative parent. The six variants were located in two sets of three consecutive exons (exons 23–25 and 46–48).

**Table 1:** *DNAH6* variants in CDGP probands.

Variant	Position on chr 2^a^	Consequence	Predicted Effect (SIFT/PolyPhen)	Family	Transmission	Global 1000G frq	SISu exome frq	CDGP frq (n variant alleles/ total alleles)	*P* ^b^
rs184604697	84926729	Stop gainedc.7689C>A, p.Tyr2563Ter	NA	Family 1	Affected parent	0.001	0.004	0	NA
rs61743118	84846930	Nonsynonymousc. 3694A>G, p.Met1232Val	Tolerated (1) / benign (0.002)	Family 2Family 5Family 6Family 11	Affected parent (Families 6, 11), other parent or de novo (Families 2, 5)	0.011	0.052	0.049 (12/246)	0.87
rs146306207	84926746	Nonsynonymousc.7706G>A, p.Arg2569His	Deleterious (0)/ probably damaging (0.958)	Family 4	Other parent or de novo	0.001	0.009	0.015 (4/270)	0.53
rs61733547	84848596	Nonsynonymousc.3992G>A, p.Arg1331His	Tolerated (0.1)/ benign (0.023)	Family 8Family 13	Affected parent (Family 8), other parent or de novo (Family 13)	0.026	0.047	0.049 (10/204)	1.0
rs114514726	84924894	Nonsynonymousc.1636G>A, p.Val546Ile	Tolerated (0.89)/ unknown	Family 8	Other parent or de novo	0.017	0.029	0.02 (5/246)	0.68
rs200844717	84928399	Nonsynonymousc.7997C>A, p.Ser2666Tyr	Deleterious (0)/ probably damaging (0.998)	Family 9Family 13	Affected parent (both)	NA	0.01	0.008 (2/252)	1.0

^a^ Position according to genome build GRCh37.

^b^ Fisher’s exact test for enrichment of allele between the Finnish SISU exome samples (*N* > 2026) and CDGP samples.

Many genes contained shared transmitted variants with no frequency annotations in the top 5 families (**S3 Table**), but little data could be gathered to prioritize any of these as plausible targets for follow-up studies. Most of the nonsynonymous variants were not predicted to be protein-damaging, and several variants were flagged as suspect or found in multiple genomic locations, suggesting that future improvements in sequence annotation is necessary before further assessment can be made. Most variants fell in *ANKRD36*, a large, frequently mutated gene.

As for regulatory variants surrounding the best functional candidate genes in the pericentromeric region of chromosome 2, nearly all of the shared variants were common or lacked publically available frequency information and could not be assessed further (**S8 Table**). For many variants, information on their potential to fall in regulatory regions was also limited, and no significant eQTLs could be detected in publically available data.

Finally, several shared variants were found in the LD block surrounding the menarche-associated variant rs6758290, but none affected protein sequence or were predicted to be important for nearby gene regulation (**S9 Table**). Five of the shared variants had frequency information available, and these were all common in the Finnish population (MAF ~0.2).

### Follow-up sequencing did not support the enrichment of DNAH6 variants in CDGP patients

Only variants annotated to *DNAH6* met sharing and frequency criteria and were predicted to alter the amino acid sequence of the protein, so these were chosen for follow-up resequencing in an additional 135 unrelated Finnish CDGP patients (**Table 1**). In our original 13 probands, we found that 10 families harbored 6 low-frequency LoF or nonsynonymous. We detected 5 of these variants in the additional CDGP samples. However, all of these variants were present in the Finnish population at similar frequencies as in the CDGP patient set. Simulation analyses showed that 10 or more out of random sets of 13 Finnish individuals frequently had LoF or nonsynonymous variants in the *DNAH6* gene (*P* = 0.13), so the sharing observed among the CDGP probands may be a chance finding. Unfortunately, there was no data available on pubertal timing for the Finnish population sample to check for delayed puberty in carriers of these variants. Only the stop variant seen in family 1 was not detected elsewhere, including the SISu sequence dataset, and thus cannot be excluded as potentially functional.

## Discussion

We hypothesized that variants in or near genes or in regulatory regions of functionally relevant candidate genes under the chromosome 2 linkage peak might harbor a shared susceptibility variant or multiple different variants disrupting the same gene or regulatory element, predisposing to a delay in puberty and growth.

Our analysis revealed that variants in *DNAH6* met our frequency and sharing criteria. In our data, the family contributing most to the previously published linkage signal transmitted a premature stop codon, and two of five other variants found in several other families were predicted to be damaging to the protein. However, when we sequenced the six exons containing these variants in an additional 135 unrelated Finnish CDGP probands, we were unable to show an enrichment of low-frequency protein-altering changes compared to the Finnish population. Additionally, although we saw a total of 10 mutations that resulted in LoF or a premature stop codon in our 13 families, simulation analyses showed that *DNAH6* often harbors mutations at similar frequency in Finnish individuals.

*DNAH6* may still be an interesting functional candidate due to its potential role in steroid hormone metabolism. This gene encodes a dynein which functions as a microtubule-associated motor protein. Dyneins can be axonemal, providing the motive force for cilia and flagella, or cytoplasmic, where they assist intercellular movement and cytoskeletal remodeling [33]. Although this particular gene is predicted by similarity-based approaches to localize in the cilium axoneme (http://www.uniprot.org/), there is a potential role for dyneins in steroid hormone metabolism via cytoskeletal trafficking of substrates [34]. Heavy chain dyneins are expressed in both the brain and testis [35]. *DNAH6* is a large gene spanning 300kb, and the largest of four isoforms is made up of 4,158 amino acid residues. Not surprising for a gene of its size, a substantial amount of variation has been documented within the gene, including 58 stop codons. The stop codon transmitted in family 1 truncates a large portion of the protein, including several ATP-binding motor subunits and the microtubule-binding stalk. The functional implication of this truncation is not known, but since this variant has not been recorded elsewhere, it cannot be ruled out.

A new locus associated with age at menarche (AAM) that falls in the region under the chromosome 2 linkage signal was recently uncovered, tagged by rs6758290 at 105.8 Mb [19]. Examining our families for shared variants in this region failed to reveal compelling evidence for low-frequency mutation that would explain the tendency toward pubertal delay. Investigation of the raw sequencing data for the SISu exome project revealed that this region of the genome is likely to be difficult to sequence, since only one variant in LD with the AAM-associated SNP could be detected with a low call-rate of 19% and an average read-depth of 2. On the other hand, the SNPs in this region were sequenced successfully in our study, with an average call depth across all families of between 13 and 54 reads per marker.

The closest gene to the menarche GWA signal is *GPR45*, a G protein-coupled receptor of which little is known. According to AceView (http://www.ncbi.nlm.nih.gov/IEB/Research/Acembly/), a BlastP search showed that the closest gene to *GPR45* in *C*. *elegans* is *npr-1*, which encodes a neuropeptide receptor which is homologous to mammalian neuropeptide Y (NPY) receptor. NPY seems to have an inhibitory effect on the hypothalamic-pituitary axis, acting as a brake which restrains the onset of puberty [36]. Furthermore, in one study, NPY levels were higher in girls with CDGP than a group of girls with normal pubertal timing [37], suggesting that it, and possibly other related receptors, may be important in pubertal delay. Thus, this region might be a good candidate for investigation in future studies, but the probands in the current study do not appear to share any low-frequency coding variants in this gene.

We also had insertion-deletion (indel) sequence data. There were many shared indel variants; for example, in family 1 there were 18 indels in candidate genes that were also shared by some of the other families (data not shown). However, the vast majority of indel variants were not annotated with allele frequency data, nor were they found in the SISu sequences, which made it very difficult to prioritize any for follow-up analysis. Further, differences in sequencing and calling methodology may affect results, and indels remain largely unannotated in terms of population frequency and function in public databases [38]. The lack of publically available population frequency information for indels, and even many SNPs, is a serious limitation in this study that may be overcome by future improvements in this area.

Our results highlight the difficulty of finding a causative mutation under a linkage finding, especially for a complex phenotype. Even with a relatively homogenous genetic background of the Finnish population [32,39], detailed phenotype data, and strict criteria for the inclusion of family members [21], analysis of CDGP families is complicated by the fact that this phenotype represents the tail of a normally distributed trait within the population, so we expect that variants which push puberty and growth toward the late end of the spectrum will also be present in the general population at a low level. Thus, we cannot use the absence of variants in the population as a filter, and instead have to assume that susceptibility variants will be enriched in patients compared to the general population. Furthermore, unknown penetrance of the susceptibility allele(s) means that unequivocal exclusion of the second parent is unwise. Additionally, our results highlight the challenge of assessing rare or low-frequency variants found in only a few individuals. While the use of a population-matched control set is critical to minimize spurious findings, even with a large resource of sequenced individuals, it can be difficult to determine which variants might be functionally relevant.

Challenges at many levels might affect our ability to detect susceptibility variants associated with CDGP. First, if the variant is subtle (i.e., regulatory) it may be very difficult to identify. While our overall sequencing coverage was good, regions that are difficult to sequence clearly exist in this region of the genome, and the causative mutation could lie in a gene that was not well-sequenced. Also, variant annotation depends on the choice of transcript set as well as the software used for annotation [40], so if variants were not annotated correctly, they may have been excluded from further analysis. Our knowledge of the regulatory regions of the genome is far from complete; thus, the causative variant might be in a regulatory region that is unknown or regulating a gene that is not an obvious functional candidate based on current knowledge.

In conclusion, the genetic variants predisposing individuals to constitutional delay of growth and puberty remain largely unknown, even when well-phenotyped samples are available from a genetically homogenous population. Future improvements in sequencing technology, genomic functional annotation, and rare variant analysis may improve our ability to analyze and interpret complex phenotypes such as CDGP.

## Supporting Information

S1 Fig Plot of the region surrounding menarche-associated variant rs6758290. The largest dark orange diamond represents rs6758290. Other variants in the region are shown. The darker and larger the diamond is, the higher the LD (r^2^) of each variant with rs6758290. The majority of variants in high LD with this SNP cluster at or upstream of *GPR45*. The LD plot was generated by the SNAP Annotation and Proxy Search Regional LD Plot tool (https://www.broadinstitute.org/mpg/snap/ldplot.php) for 1000 Genomes Pilot 1 SNPs in the CEU reference population.
(TIF)

S1 Table Genotyping results. (DOCX)

S2 Table Genic variants transmitted from the affected parent with 1000G frequencies of <5%. (DOCX)

S3 Table Genic variants transmitted from the affected parent with no population frequency data available in 1000 Genomes. (DOCX)

S4 Table Variants annotated to any gene and with 1000G frequencies available (< 5%), with transmission from either parent. (DOCX)

S5 Table GO terms queried for candidate genes in the pericentromeric region of chr 2. (DOCX)

S6 Table Candidate genes annotated with appropriate GO terms on chromosome 2 between 79–124 Mb. (DOCX)

S7 Table Genes and gene boundaries queried in the regulatory region analysis. (DOCX)

S8 Table Regulatory region analysis surrounding the top 8 functional candidate genes. (DOCX)

S9 Table Investigation of the menarche-associated locus surrounding rs6758290. (DOCX)

S10 Table Primer sequences for *DNAH6* exons (5’ to 3’). (DOCX)

S11 Table A comparison of average genotype quality and read-depth for *DNAH6* variants between SISu exomes (*N* = 2,028) and CDGP probands (*N* = 13). (DOCX)
