# Supplementary figures and images for: Targeted Resequencing of the Pericentromere of Chromosome 2 Linked to Constitutional Delay of Growth and Puberty
Source: PLoS One. 2015 Jun 1;10(6):e0128524. doi: 10.1371/journal.pone.0128524 (PMC4452275; doi:10.1371/journal.pone.0128524)

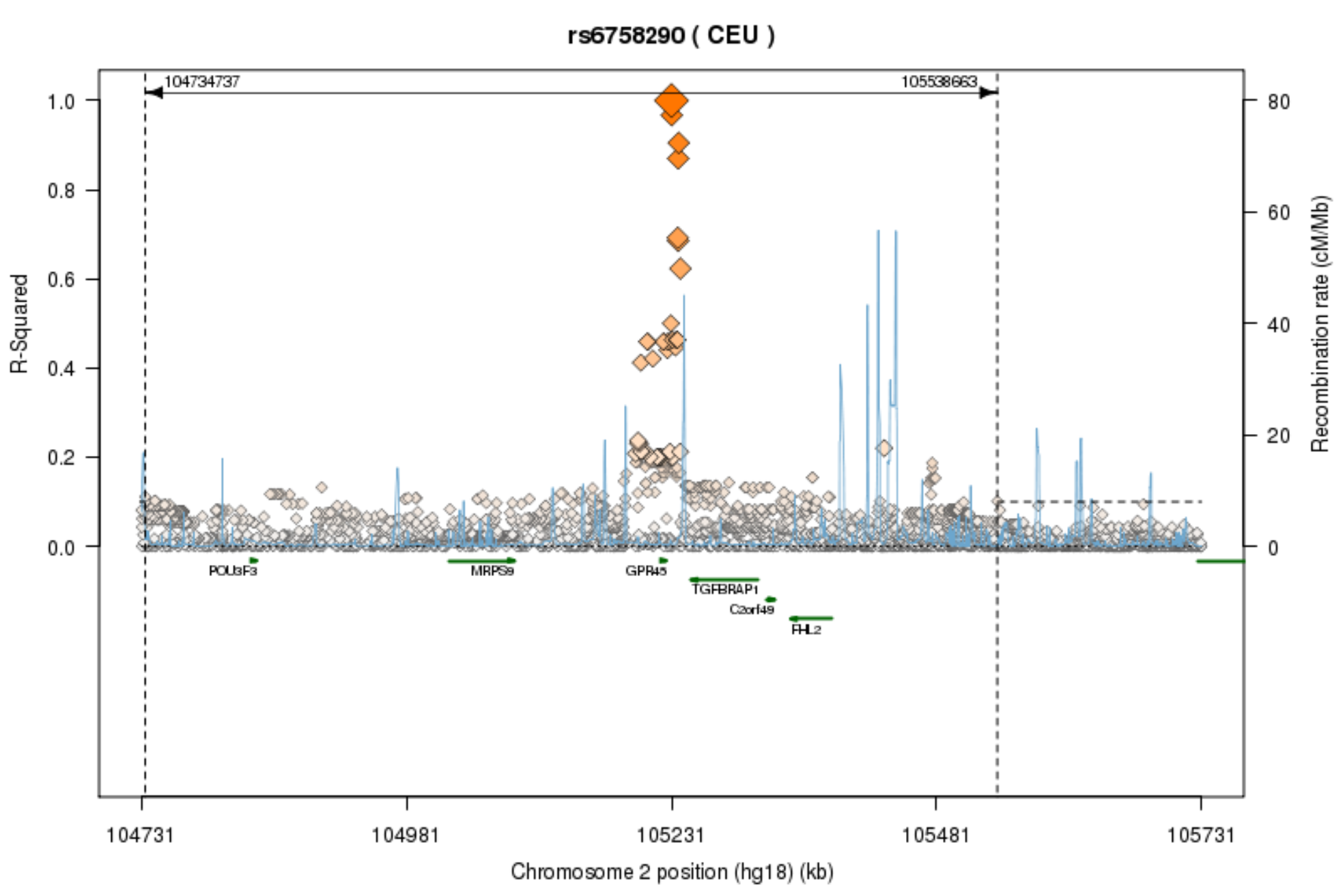

Supplement: S1 Fig — The largest dark orange diamond represents rs6758290. Other variants in the region are shown. The darker and larger the diamond is, the higher the LD (r2) of each variant with rs6758290. The majority of variants in high LD with this SNP cluster at or upstream of GPR45. The LD plot was generated by the SNAP Annotation and Proxy Search Regional LD Plot tool (https://www.broadinstitute.org/mpg/snap/ldplot.php) for 1000 Genomes Pilot 1 SNPs in the CEU reference population. (TIF) [file pone.0128524.s001.tif]
