# Supplementary material for: Targeted Resequencing of the Pericentromere of Chromosome 2 Linked to Constitutional Delay of Growth and Puberty
Source: PLoS One. 2015 Jun 1;10(6):e0128524. doi: 10.1371/journal.pone.0128524 (PMC4452275; doi:10.1371/journal.pone.0128524)
Supplement: S1 Table — (DOCX) [file pone.0128524.s002.docx]

**Table S1. Genotyping results.** Three rounds of genotyping were performed, with 8 samples genotyped on the HD HumanCNV370-Quad Beadchip (Illumina) and the remaining 30 on the Human610-Quad Beadchip (Illumina), in two batches. CNVs appeared as failed genotypes, thus contributing to the low success rate estimated for that batch. † CNVs included. ‡ CNVs excluded.

| **Chromosome 2, 67-130 Mb** | | | | | |
| --- | --- | --- | --- | --- | --- |
| **Platform** | **Samples genotyped** | **N samples** | **N SNPs** | **Genotyping success rate** | **Average intermarker distance (bp)** |
| **HD HumanCNV370-Quad Beadchip** | Trios 1 and 2  Probands 12 and 13 | 8 | 6313 | 0.96 | 9979 |
| **Human610-Quad Beadchip** | Trios 3, 4, 5, and 6  Parents 13 | 14 | 11 155† | 0.93 | 5647 |
| **Human610-Quad Beadchip** | Trios 7, 8, 9, and 10  Parents 12  Affected parent and proband 11 | 16 | 10 710‡ | 0.97 | 5881 |
