# Supplementary material for: Targeted Resequencing of the Pericentromere of Chromosome 2 Linked to Constitutional Delay of Growth and Puberty
Source: PLoS One. 2015 Jun 1;10(6):e0128524. doi: 10.1371/journal.pone.0128524 (PMC4452275; doi:10.1371/journal.pone.0128524)
Supplement: S2 Table — (DOCX) [file pone.0128524.s003.docx]

**Table S2. Genic variants transmitted from the affected parent with 1000G frequencies of <5%.**

| **Gene** | **Variant** | **Position (GRCh37)** | **Allele** | **Family** | **EUR MAF (1000G)^a^** | **Exome Variant Server MAF^b^** | **FIN MAF (1000G)^c^** | **Expanded FIN MAF**  **(SISU)^d^** | **Consequence** | **SIFT^e^** | **Polyphen^e^** | **Regulome score^f^** |
| --- | --- | --- | --- | --- | --- | --- | --- | --- | --- | --- | --- | --- |
| *DNAH6* | rs184604697 | 84926729 | A | 1 | 0.004 | Variant not found; avg. seq depth in Eur samples = 67 | 0.016 | 0.004 | STOP gained | No data | No data | NA |
| *DNAH6* | rs61743118 | 84846930 | G | 6, 12 | 0.029 | 0.015 | 0.048 | 0.05 | Met1232Val | tolerated (1) | benign (0.002) | NA |
| *DNAH6* | rs61733547 | 84848596 | A | 8 | 0.058 | 0.043 | 0.065 | 0.047 | Arg1331His | tolerated (0.1) | probably damaging (0.947) | NA |
| *DNAH6* | rs200844717 | 84928399 | A | 9, 13 | No data | 0.007 | No data | 0.01 | Ser2666Tyr | deleterious (0) | probably damaging (1) | NA |
| *KDM3A* | rs55781109 | 86714555 | G | 1 | 0.017 | NA | 0.022 | NA | intronic | NA | NA | No data |
| *KDM3A* | rs77936006 | 86718519 | T | 8 | 0.046 | NA | 0.022 | NA | intronic | NA | NA | 6 |
| *KDM3A* | rs72844700 | 86706134 | G | 3 | 0.037 | NA | 0.016 | NA | intronic | NA | NA | 6 |
| *KDM3A* | rs150660157 | 86706188 | C | 12 | 0.009 | NA | 0.011 | NA | intronic | NA | NA | no data |
| *KDM3A* | rs182941106 | 86709906 | T | 13 | 0.003 | NA | 0.011 | NA | intronic | NA | NA | no data |
| *LINC01102* | rs72828213 | 105124035 | T | 1, 3, 9 | 0.013 | NA | 0.043 | NA | intronic | NA | NA | 6 |
| *LINC01102* | rs148208172 | 105050639 | C | 1 | 0.013 | NA | 0.054 | NA | upstream | NA | NA | no data |
| *LINC01102* | rs191128853 | 105123170 | A | 7 | 0.003 | NA | 0.005 | NA | intronic | NA | NA | no data |
| *LINC01102* | rs115505403 | 105137155 | C | 11 | 0.028 | NA | 0.022 | NA | downstream | NA | NA | 6 |

^a^The minor allele frequency from the European subset of samples in the 1000 Genomes release 14 (<http://browser.1000genomes.org/index.html>).

^b^European samples only. Exome Variant Server, NHLBI GO Exome Sequencing Project (ESP), Seattle, WA (URL: http://evs.gs.washington.edu/EVS/) [June 2014].

^c^The minor allele frequency from the Finnish subset of samples in the 1000 Genomes release 14.

^d^The minor allele frequency from the Finnish samples in the SISU Finnish exome database (<http://sisuproject.fi/>).

^e^SIFT and POLYPHEN predictions taken from the Variant Effect Predictor (<http://www.ensembl.org/info/docs/tools/vep/index.html>) for genome build GRCh37.

^f^See RegulomeDB website for detailed scoring information (<http://regulome.stanford.edu/index>).
