# Supplementary material for: Targeted Resequencing of the Pericentromere of Chromosome 2 Linked to Constitutional Delay of Growth and Puberty
Source: PLoS One. 2015 Jun 1;10(6):e0128524. doi: 10.1371/journal.pone.0128524 (PMC4452275; doi:10.1371/journal.pone.0128524)
Supplement: S3 Table — (DOCX) [file pone.0128524.s004.docx]

**Table S3. Genic variants transmitted from the affected parent with no population frequency data available in 1000 Genomes.**

| **Gene** | **Variant** | **Position (GRCh37)** | **Variant allele** | **Family** | **Exome Variant Server MAF^a^** | **FIN MAF (SISU)^b^** | **Consequence** | **SIFT^c^** | **Polyphen^c^** | **Regulome Score^d^** |
| --- | --- | --- | --- | --- | --- | --- | --- | --- | --- | --- |
| ANKRD36C | rs5005867 | 96521336 | C | 1, 2, 3, 4, 5, 6, 7, 8, 10, 11, 12, 13 | not found | not found | Nonsynonymous codon | Tolerated (0.16) | Possibly damaging (0.617, 0.892) | no data |
| ANKRD36C | rs5005868 | 96521324 | G | 1, 2, 3, 4, 5, 6, 7, 8, 10, 11, 12, 13 | not found | not found | Nonsynonymous codon | Tolerated (0.09) | Probably/ possibly damaging (0.711, 0.926) | 6 |
| ANKRD36C | rs62154561 | 96604591 | G | 1, 2, 3, 4, 5, 6, 7, 8, 9, 10, 11, 12, 13 | not found | not found | Nonsynonymous codon | Tolerated (0.14) | Benign (0.001) | no data |
| AC073995.2 | rs62154618 | 96619734 | T | 1, 2, 3, 4, 5, 6, 7, 8, 9, 10, 11, 12, 13 | not found | not found | Nonsynonymous codon | tolerated | possibly damaging (0.494) | no data |
| ANKRD36C | rs71186754 | 96521334 | C | 1 | not found | not found | Nonsynonymous codon | Tolerated (0.37) | Possibly damaging (0.465) | no data |
| ANKRD36C | rs74755605 | 96517518 | G | 1, 2, 3, 4, 5, 6, 7, 8, 9, 10, 11, 12, 13 | not found | not found | Nonsynonymous codon | Tolerated (1) | Probably damaging (1) | no data |
| ANKRD36C | rs74946480 | 96619688 | G | 1, 2, 3, 4, 5, 6, 7, 8, 9, 10, 11, 12, 13 | not found | not found | Nonsynonymous codon | Tolerated (0.11) | Benign (0.002) | no data |
| ANKRD36C | rs76384706 | 96517484 | G | 1, 2, 3, 4, 8, 10, 13 | not found | not found | Nonsynonymous codon | Tolerated (1) | Benign (0) | no data |
| ANKRD36C | rs78179792 | 96616504 | A | 1, 2, 3, 4, 5, 6, 7, 8, 9, 10, 11, 12, 13 | not found | not found | Nonsynonymous codon | Tolerated (0.1) | Benign (0.023) | no data |
| ANKRD36C | rs79392443 | 96517502 | C | 1, 2, 3, 4, 5, 6, 7, 8, 9, 10, 12, 13 | not found | not found | Nonsynonymous codon | Deleterious (0.01) | Possibly damaging (1) | 6 |
| ANKRD36C | rs80041617 | 96517905 | T | 1, 2, 3, 6, 7, 8, 9, 10 | not found | not found | Nonsynonymous codon | Tolerated (0.13) | Probably damaging (1) | no data |
| ANAPC1 | rs79100806 | 112615888 | G | 1, 9, 10 | not found | not found | Nonsynonymous codon | Tolerated (0.11) | Benign (0.002) | no data |
| ANKRD36 | rs10194525 | 97877478 | A | 1, 2, 3, 4, 5, 6, 7, 9, 10, 11, 12 | not found | not found | Nonsynonymous codon | Deleterious (0.01) | Probably damaging (0.995) | no data |
| ANKRD36 | rs112133169 | 97833466 | T | 1, 2, 3, 4, 5, 6, 7, 8, 9, 10, 11, 12, 13 | not found | not found | Nonsynonymous codon | Tolerated (0.2) | Benign (0.349) | no data |
| ANKRD36 | rs35711845 | 97877440 | C | 1, 2, 6, 7, 8, 9, 10, 11, 12 | 0.497 | not found | Nonsynonymous codon | Deleterious (0.01) | Benign (0.024) | 6 |
| ANKRD36 | rs5008284 | 97833342 | A | 1, 2, 3, 4, 5, 6, 7, 8, 9, 10, 11, 12, 13 | NA | NA | Splice region variant | NA | NA | no data |
| ANKRD36 | rs59466168 | 97860487 | C | 1, 2, 3, 4, 5, 7, 8, 9, 10, 11, 12, 13 | not found | not found | Nonsynonymous codon | Tolerated (0.21) | Probably damaging (1) | no data |
| ANKRD36 | rs60795069 | 97864374 | T | 1, 2, 3, 4, 5, 6, 7, 8, 9, 10, 11, 12, 13 | not found | not found | Nonsynonymous codon | Tolerated (0.06) | Benign (0.001) | 6 |
| ANKRD36 | rs62153044 | 97808394 | G | 1, 2, 3, 4, 5, 6, 7, 8, 9, 10, 11, 12, 13 | not found | not found | Nonsynonymous codon | Tolerated (0.19) | Possibly damaging (0.539) | 6 |
| ANKRD36BP2 | rs76265890 | 89104928 | C | 1, 6, 7, 8, 10, 11, 12, 13 | NA | NA | Splice region variant | NA | NA | no data |
| IGKV1D-13 | rs1724244 | 90193417 | G | 1, 8 | not found | not found | Nonsynonymous codon | Tolerated (1) | Benign (0) | 6 |
| IGKV1D-8 | rs58787496 | 90259607 | T | 1, 6, 11, 12 | NA | NA | 5' UTR variant | NA | NA | 4 |
| IGKV1D-8 | rs60708916 | 90259643 | C | 1, 2, 6, 7, 8, 9, 10, 11, 12, 13 | NA | NA | 5' UTR variant | NA | NA | 4 |
| IGKV1D-8 | rs75674860 | 90259717 | A | 1, 2, 6, 7, 8, 9, 10, 11, 12, 13 | NA | NA | 5' UTR variant | NA | NA | 4 |
| RETSAT | rs4832168 | 85570849 | T | 1 | not found | not found | Nonsynonymous codon | deleterious (0) | probably damaging (1) | 5 |

^a^European samples only. Exome Variant Server, NHLBI GO Exome Sequencing Project (ESP), Seattle, WA (URL: http://evs.gs.washington.edu/EVS/) [June 2014].

^b^The minor allele frequency from the Finnish subset of samples in the 1000 Genomes release 14.

^c^SIFT and POLYPHEN predictions taken from the Variant Effect Predictor (<http://www.ensembl.org/info/docs/tools/vep/index.html>) for genome build GRCh37.

^d^See RegulomeDB website for detailed scoring information (<http://regulome.stanford.edu/index>).
