# Supplementary material for: Targeted Resequencing of the Pericentromere of Chromosome 2 Linked to Constitutional Delay of Growth and Puberty
Source: PLoS One. 2015 Jun 1;10(6):e0128524. doi: 10.1371/journal.pone.0128524 (PMC4452275; doi:10.1371/journal.pone.0128524)
Supplement: S4 Table — (DOCX) [file pone.0128524.s005.docx]

**Table S4. Variants annotated to any gene and with 1000G frequencies available (< 5 %), with transmission from either parent.**

| **Gene** | **Variant** | **Position (GRCh37)** | **Variant allele** | **Family** | **1000G Eur frq^a^** | **FIN 1000G frq^b^** | **Consequence** | **Transmission** | **SIFT^c^** | **PolyPhen^c^** |
| --- | --- | --- | --- | --- | --- | --- | --- | --- | --- | --- |
| *DDX18* | rs61748152 | 118578770 | A | 1 | 0.0073 | 0.011 | Non-synonymous variant | affected parent | Deleterious (0.03) | Benign (0.007) |
|  | rs61755349 | 118582189 | A | 1 | 0.0064 | 0.011 | Non-synonymous variant | affected parent | Tolerated (0.11) | Benign (0.007) |
|  | rs1052639 | 117825524 | A | 2 | 0.0384 | 0.081 | Synonymous variant | unaffected parent or de novo | NA | NA |

^a^ The minor allele frequency from the European subset of samples in the 1000 Genomes release 14.

^b^The minor allele frequency from the Finnish subset of samples in the 1000 Genomes release 14.

^c^SIFT and POLYPHEN predictions taken from the Variant Effect Predictor (<http://www.ensembl.org/info/docs/tools/vep/index.html>) for genome build GRCh37
