# Supplementary material for: Targeted Resequencing of the Pericentromere of Chromosome 2 Linked to Constitutional Delay of Growth and Puberty
Source: PLoS One. 2015 Jun 1;10(6):e0128524. doi: 10.1371/journal.pone.0128524 (PMC4452275; doi:10.1371/journal.pone.0128524)
Supplement: S5 Table — (DOCX) [file pone.0128524.s006.docx]

**Table S5. GO terms queried for candidate genes in the pericentromeric region of chr 2.** Gene Ontology terms were queried using the Amigo search tool (<http://amigo1.geneontology.org/cgi-bin/amigo/go.cgi>).

adenohypophysis development

adenohypophysis formation

adenohypophysis morphogenesis

cellular process involved in reproduction in multicellular organism

development of primary sexual characteristics

development of secondary sexual characteristics

developmental process involved in reproduction

gonad development

gonad morphogenesis

gonadotrophin-releasing hormone neuronal migration to the hypothalamus

gonadotropin-releasing hormone binding

growth hormone activity

hypophysis formation

hypophysis morphogenesis

hypothalamus cell differentiation

hypothalamus cell migration

hypothalamus development

hypothalamus gonadotrophin-releasing hormone neuron development

hypothalamus gonadotrophin-releasing hormone neuron differentiation

hypothalamus gonadotrophin-releasing hormone neuron fate commitment

kisspeptin receptor binding

multicellular organism reproduction

positive regulation of gonadotropin secretion

regulation of female gonad development

regulation of gonadotropin secretion

regulation of male gonad development

reproduction

response to gonadotropin stimulus

sexual reproduction

negative regulation of gonadotropin secretion

cellular response to gonadotropin stimulus

response to gonadotropin-releasing hormone

gonadotropin hormone-releasing hormone activity

gonadotropin-releasing hormone receptor binding

gonadotropin-releasing hormone receptor activity

hypothalamus gonadotrophin-releasing hormone neuron development

hypothalamus gonadotrophin-releasing hormone neuron fate commitment

hypothalamus gonadotrophin-releasing hormone neuron differentiation

gonadotrophin-releasing hormone neuronal migration to the hypothalamus

luteinizing hormone receptor activity

luteinizing hormone secretion

luteinizing hormone signaling pathway

response to luteinizing hormone stimulus

regulation of luteinizing hormone secretion

follicle-stimulating hormone complex

follicle-stimulating hormone activity

follicle-stimulating hormone secretion

follicle-stimulating hormone receptor binding

follicle-stimulating hormone receptor activity

follicle-stimulating hormone signaling pathway

response to follicle-stimulating hormone stimulus

regulation of follicle-stimulating hormone secretion

cellular response to follicle-stimulating hormone stimulus

menarche

growth

regulation of growth

developmental growth involved in morphogenesis

developmental growth

growth factor binding

growth factor activity

growth hormone activity

growth hormone secretion

growth hormone receptor binding

growth factor receptor binding

growth hormone receptor complex

growth hormone receptor activity

insulin-like growth factor binding

response to growth factor stimulus

regulation of developmental growth

response to growth hormone stimulus
