# Supplementary material for: Targeted Resequencing of the Pericentromere of Chromosome 2 Linked to Constitutional Delay of Growth and Puberty
Source: PLoS One. 2015 Jun 1;10(6):e0128524. doi: 10.1371/journal.pone.0128524 (PMC4452275; doi:10.1371/journal.pone.0128524)
Supplement: S6 Table — (DOCX) [file pone.0128524.s007.docx]

**Table S6. Candidate genes annotated with appropriate GO terms on chromosome 2 between 79-124 Mb.** Genes marked in bold are considered the best candidate genes which are annotated to both growth and reproductive functions, or direct involvement in the hypothalamic-pituitary-gonadal axis.

| **Gene** | **Position (Mb) on chr 2** | **General Function (Aceview)** | **GO term(s)** |
| --- | --- | --- | --- |
| ADRA2B | 96.7 | G protein-coupled receptor. Critical role in regulating neurotransmitter release from sympathetic nerves and from adrenergic neurons in the central nervous system. Associated with reduced basal metabolic rate in obese subjects | Reproduction |
| ASTL | 96.7 | Proteolysis, metalloendopeptidase | Reproduction, sexual reproduction |
| **BCL2L11** | **111.8** | **Apoptotic activator** | **Development of primary sexual characteristics, developmental process involved in reproduction, gonad development, multicellular organism reproduction, reproduction, sexual reproduction, regulation of growth, response to growth factor stimulus** |
| EIF2AK3 | 88.8 | Phosphorylates the alpha subunit of eukaryotic translation-initiation factor 2 (EIF2), leading to its inactivation, and thus to a rapid reduction of translational initiation and repression of global protein synthesis | Multicellular organism reproduction, reproduction |
| EPB41L5 | 120.7 | Embryonic development, response to TGF-beta stimulus | Growth, developmental growth involved in morphogenesis |
| FOXD4L1 | 114.2 | Embryogenesis, regulation of transcription | Growth, developmental growth involved in morphogenesis, developmental growth |
| **GLI2** | **121.5** | **Mediator of Sonic hedgehog (Shh) signaling, embryogenesis including pituitary development** | **Developmental process involved in reproduction, reproduction, growth, developmental growth** |
| IL1A | 113.5 | Pleiotropic cytokine involved in various immune responses, inflammatory processes, and hematopoiesis | Growth factor receptor binding |
| **IL1B** | **113.5** | **Cell proliferation, differentiation, and apoptosis** | **Reproduction, growth factor activity, growth factor receptor binding** |
| IL1F10 | 113.8 | Network of interleukin 1 family members to regulate adapted and innate immune responses | Growth factor receptor binding |
| IL1R1 | 102.7 | mediator involved in many cytokine induced immune and inflammatory responses | Growth factor receptor binding |
| IL1RN | 113.8 | Increased risk of osteoporotic fractures and gastric cancer | Reproduction, growth factor receptor binding |
| IL36A | 113.7 | Interleukin 1 cytokine family, ankylosing spondylitis, spondylitis | Growth factor receptor binding |
| IL36B | 113.7 | Stimulates production of interleukin-6 and interleukin-8 in synovial fibrobasts, articular chondrocytes and mature adipocytes, ankylosing spondylitis, spondylitis | Growth factor receptor binding |
| IL36G | 113.7 | Interleukin 1 cytokine family; herpes simplex and ankylosing spondylitis | Growth factor receptor binding |
| IL36RN | 113.8 | Interleukin 1 cytokine family; generalized pustular psoriasis | Growth factor receptor binding |
| IL37 | 113.7 | Interleukin 1 cytokine family; morbid obesity and ankylosing spondylitis | Growth factor receptor binding |
| **INHBB** | **121.1** | **Inhibin beta B subunit joins the alpha subunit to form a pituitary FSH secretion inhibitor** | **Cellular process involved in reproduction in multicellular organism, development of primary sexual characteristics, developmental process involved in reproduction, gonad development, multicellular organism reproduction, positive regulation of gonadotropin secretion, regulation of gonadotropin secretion, reproduction, sexual reproduction, negative regulation of gonadotropin secretion, regulation of follicle-stimulating hormone secretion, growth, growth factor activity** |
| **KDM3A** | **86.6** | **Hormone-dependent transcriptional activation** | **Cellular process involved in reproduction in multicellular organism, developmental process involved in reproduction, multicellular organism reproduction, reproduction, sexual reproduction** |
| LIMS1 | 109.1 | Growth factor receptor kinase signaling pathways | Response to growth factor stimulus |
| **MERTK** | **112.6** | **Receptor tyrosine kinase that transduces signals from the extracellular matrix into the cytoplasm by binding to several ligands. Induces phosphorylation of MAPK1. Autosomal recessive retinitis pigmentosa** | **Development of primary sexual characteristics, developmental process involved in reproduction, multicellular organism reproduction, reproduction, sexual reproduction** |
| **NPHP1** | **110.9** | **Control of cell division, as well as in cell-cell and cell-matrix adhesion signaling, likely as part of a multifunctional complex localized in actin- and microtubule-based structures** | **Cellular process involved in reproduction in multicellular organism, developmental process involved in reproduction, multicellular organism reproduction, reproduction, sexual reproduction** |
| **PAX8** | **113.9** | **Thyroid follicular cell development and expression of thyroid-specific genes** | **Response to gonadotropin stimulus, cellular response to gonadotropin stimulus** |
| RALB | 121 | GTP-binding protein that belongs to the small GTPase superfamily and Ras family of proteins | Response to growth factor stimulus |
| REG1A | 79.3 | Islet cell regeneration and diabetogenesis and may be involved in pancreatic lithogenesis | Growth factor activity |
| ST6GAL2 | 107.4 | Protein amino acid glycolysation | Growth |
| TFCP2L1 | 121.9 | Cell morphogenesis, positive regulation of growth, hypothyroidism | Reproduction, regulation of growth |
| TGFBRAP1 | 105.8 | Transforming growth factor beta receptor signaling pathway | Response to growth factor stimulus |
| TSGA10 | 99.6 | Spermatogenesis | Multicellular organism reproduction, reproduction, sexual reproduction, |
| TTL | 113.2 | Cytosolic enzyme involved in the posttranslational modification of alpha-tubulin | Regulation of growth, regulation of developmental growth |
