# Supplementary material for: Targeted Resequencing of the Pericentromere of Chromosome 2 Linked to Constitutional Delay of Growth and Puberty
Source: PLoS One. 2015 Jun 1;10(6):e0128524. doi: 10.1371/journal.pone.0128524 (PMC4452275; doi:10.1371/journal.pone.0128524)
Supplement: S7 Table — (DOCX) [file pone.0128524.s008.docx]

**Table S7.** **Genes and gene boundaries queried in the regulatory region analysis**. (Genome build GRCh37)

| **Gene** | **-200 Kb** | **+200Kb** |
| --- | --- | --- |
| *KDM3A* | 86467770 | 86919839 |
| *PAX8* | 113773574 | 114236527 |
| *INHBB* | 120903719 | 121309384 |
| *GLI2* | 121293199 | 121950229 |
| *NPHP1* | 110679888 | 111162643 |
| *BCL2L11* | 111676955 | 112126024 |
| *MERTK* | 112456056 | 112987138 |
| *IL1B* | 113387328 | 113794480 |
