# Supplementary material for: Targeted Resequencing of the Pericentromere of Chromosome 2 Linked to Constitutional Delay of Growth and Puberty
Source: PLoS One. 2015 Jun 1;10(6):e0128524. doi: 10.1371/journal.pone.0128524 (PMC4452275; doi:10.1371/journal.pone.0128524)
Supplement: S8 Table — (DOCX) [file pone.0128524.s009.docx]

**Table S8.** **Regulatory region analysis surrounding the top 8 functional candidate genes.**

| **Gene** | **Position** | **Variant** | **Transmitted from affected parent** | **Allele** | **Frequency** | **RegulomeDB Score** | **Notes** |
| --- | --- | --- | --- | --- | --- | --- | --- |
| *NPHP1* | 111149445 | rs4953813 | 1, 2, 3, 4, 5, 6, 7, 8, 9, 10 | W | unknown | no data |  |
| *BCL2L11* | 112044065 | rs71431175 | 1, 2, 3, 4, 5, 6, 8, 9, 10, 12, 13 | R | unknown | 6 |  |
| *MERTK* | 112718733 | no snp | 1, 2, 3, 4 | Y | unknown | 6 |  |
| *IL1B* | 113511103 | no snp | 1, 2, 3, 4, 10, 12 | Y | unknown | no data |  |
| *IL1B* | 113558058 | rs72126246 | 1, 2, 3, 4, 7, 9, 10, 11, 12, 13 | Y |  | 6 | Overlaps large deletion |
| *INHBB* | 121148965 | rs55792386 | 1, 2, 3, 4, 6, 7, 8, 9, 10, 11 | M | unknown | 5 |  |
| *GLI2* | 121446302 | rs11122808 | 1, 2, 3, 4, 5, 6, 7, 8, 9, 11, 12, 13 | C | unknown | 6 | Whole region appears poorly sequenced in reference genomes and possibly complicated by CNVs |
| *GLI2* | 121782275 | no snp | 1, 2, 3, 4, 5, 6, 7, 8, 9, 10, 11, 13 | T | unknown | no data |  |
| *GLI2* | 121782302 | no snp | 1, 2, 3, 4, 5, 6, 7, 8, 9, 10, 11, 13 | T | unknown | no data |  |
| *GLI2* | 121782313 | no snp | 1, 2, 3, 4, 5, 6, 7, 9, 10 | R | unknown | no data |  |
