# Supplementary material for: Targeted Resequencing of the Pericentromere of Chromosome 2 Linked to Constitutional Delay of Growth and Puberty
Source: PLoS One. 2015 Jun 1;10(6):e0128524. doi: 10.1371/journal.pone.0128524 (PMC4452275; doi:10.1371/journal.pone.0128524)
Supplement: S9 Table — (DOCX) [file pone.0128524.s010.docx]

**Table S9.** **Investigation of the menarche-associated locus surrounding rs6758290.** We extracted all variants within the LD block (r^2^≥0.1) surrounding rs6758290 (105,364,800 – 106,164,800 along chr 2) and looked for any transmitted shared variants.

| **Position (GRCh37)** | **Variant** | **Variant allele** | **Family** | **Frequency** | **Consequence** | **RegulomeDB Score** |
| --- | --- | --- | --- | --- | --- | --- |
| 105563616 | no snp | R | 1, 2, 6 , 9, 11, 13 | unknown | No data | no data |
| 105671987 | rs6717222 | T | 1, 2, 3, 6, 7, 8, 9, 11, 13 | unknown; Venter is heterozygote | Intron variant | no data |
| 105878106 | rs6745194 | A | 1, 2, 7, 8, 10, 12 | FIN frq 0.18 | 5kb downstream | 6 |
| 105882780 | rs11685266 | T | 1, 2, 7, 8, 10, 12 | FIN frq 0.19 | 3’ UTR | 5 |
| 105896322 | rs13011694 | T | 1, 2, 7, 8, 10, 12 | FIN frq 0.18 | Intron variant | no data |
| 105969092 | rs34901603 |  | 1, 2, 7, 8, 10, 12 | FIN frq 0.19 | 5kb downstream | no data |
| 105973995 | rs35202975 |  | 1, 2, 7, 8, 10, 12 | FIN frq 0.2 | 5kb downstream | 5 |
