# Supplementary material for: Targeted Resequencing of the Pericentromere of Chromosome 2 Linked to Constitutional Delay of Growth and Puberty
Source: PLoS One. 2015 Jun 1;10(6):e0128524. doi: 10.1371/journal.pone.0128524 (PMC4452275; doi:10.1371/journal.pone.0128524)
Supplement: S10 Table — (DOCX) [file pone.0128524.s011.docx]

**Table S10. Primer sequences for *DNAH6* exons (5’ to 3’).**

| 23F | CCATGACCAGTATAATTG | 23R | TATGCTTAGAGTGAGAAT |
| --- | --- | --- | --- |
| 24F | ATAGTGGAATGTCAATAG | 24R | ATGTTTCTTAAATATGTGAT |
| 25F | GTAACTCACACTCACATT | 25R | TGTCAGAGCATTAGAATT |
| 46F | TTGCTATGTTAGAACTTC | 46R | AATACAAAGGAAACCAAT |
| 47F | TATCTACTATGCTGACAT | 47R | TCTCTATATGAATAAATTCCT |
| 48F | TTATTGAAATGACACAAC | 48R | GAGAATGGACTAATACAG |
