# Supplementary material for: Targeted Resequencing of the Pericentromere of Chromosome 2 Linked to Constitutional Delay of Growth and Puberty
Source: PLoS One. 2015 Jun 1;10(6):e0128524. doi: 10.1371/journal.pone.0128524 (PMC4452275; doi:10.1371/journal.pone.0128524)
Supplement: S11 Table — (DOCX) [file pone.0128524.s012.docx]

**Table S11. A comparison of average genotype quality and read-depth for *DNAH6* variants between SISu exomes (*N* = 2,028) and CDGP probands (*N* = 13).** For the CDGP probands, only biallelic variants were included in the calculation.

|  | **Average genotype quality** | **Average read-depth** |
| --- | --- | --- |
| **SISu exome set** | 83.67 | 55 |
| **CDGP probands** | 91.9 | 49 |
